# A Rare Case of Testicular Vein Thrombosis in an 18-Year-Old Male

**DOI:** 10.7759/cureus.98988

**Published:** 2025-12-11

**Authors:** Mariyam Khan, Muhammad Irfan, Kevin Xuan Hong Tang

**Affiliations:** 1 General Surgery, Royal Hampshire County Hospital, Winchester, GBR; 2 Urology, Royal Hampshire County Hospital, Winchester, GBR

**Keywords:** acute scrotal swelling, idiopathic thrombosis, scrotal pain, testicular vein thrombosis, therapeutic anticoagulation

## Abstract

Testicular vein thrombosis is a rare cause of acute scrotal pain and swelling, with very few cases reported in adolescents. We present a case of an 18-year-old male with a 24 h history of sudden-onset left scrotal pain and swelling, without prior trauma, infection, or surgery. Physical examination revealed mild tenderness along the spermatic cord, and Doppler ultrasound demonstrated a non-compressible thrombus in the left testicular vein extending into the abdomen, confirming the diagnosis. The patient was treated with a short four-week course of apixaban on hematology advice, along with activity restriction and scrotal support. Follow-up imaging showed partial recanalization and complete symptomatic improvement. This case underscores the importance of early recognition, Doppler imaging, and conservative anticoagulation therapy in achieving favorable outcomes while avoiding unnecessary surgical exploration.

## Introduction

Spontaneous thrombosis of the testicular vein is a rare cause of acute scrotal pain and swelling, particularly in young patients. While a few cases have been reported in adolescents, most documented cases occur in adults [1-4]. It can clinically mimic more common causes of acute scrotal pain, such as epididymo-orchitis, testicular torsion, or varicocele, which makes early recognition challenging [2,3,5]. Although trauma, infection, surgery, or systemic disease may predispose to venous thrombosis, most cases remain idiopathic, with no identifiable risk factors [4-7]. Doppler ultrasound is the first-line imaging modality, allowing direct visualization of thrombus, assessment of venous flow, and monitoring during conservative management, while CT angiography can evaluate thrombus extension into the abdomen [2,3,8].

Management is typically conservative and includes short-term anticoagulation when indicated, activity modification, and scrotal support. Early recognition is essential to guide appropriate treatment and prevent unnecessary surgical intervention. Awareness of testicular vein thrombosis in adolescents and young adults helps clinicians distinguish it from other causes of scrotal pain, plan follow-up imaging to confirm thrombus resolution, and ensure optimal outcomes [1,4,7,8]. This study highlights the importance of maintaining a high index of suspicion and using timely imaging to achieve successful conservative management of this rare condition.

## Case presentation

An 18-year-old male presented with a 24 h history of left scrotal pain and swelling. There was no history of trauma, infection, surgery, or vigorous activity. Examination revealed mild tenderness along the spermatic cord and slight swelling of the left hemiscrotum. Laboratory investigations for thrombophilia were largely unremarkable, except for a mildly raised β2-glycoprotein IgG, which hematology considered clinically insignificant (Table 1).

**Table 1:** Hematological investigations performed for thrombophilia screening.

Test parameters	Results	Reference range	Units
IgG anticardiolipin antibodies	6.2	0-17.8	U/mL
IgM anticardiolipin antibodies	2.7	0-10.4	U/mL
Dilute Russell's viper venom time (dRVVT)	1.09	0.85-1.15	Ratio
Dilute activated partial thromboplastin time (dAPTT)	1.09	0.87-1.24	Ratio
Prothrombin time (PT)	14.0	12.4-14.6	s
Activated partial thromboplastin time (APTT)	28.9	26.3-37.7	s
Fibrinogen	2.5	1.89-4.3	g/L
β2-glycoproteins IgM	0.6	0-6.6	U/mL
β2-glycoproteins IgG	15.8	0-10	U/mL
Antithrombin activity	119.2	81.4-126.6	IU/dL

**Figure 1:**
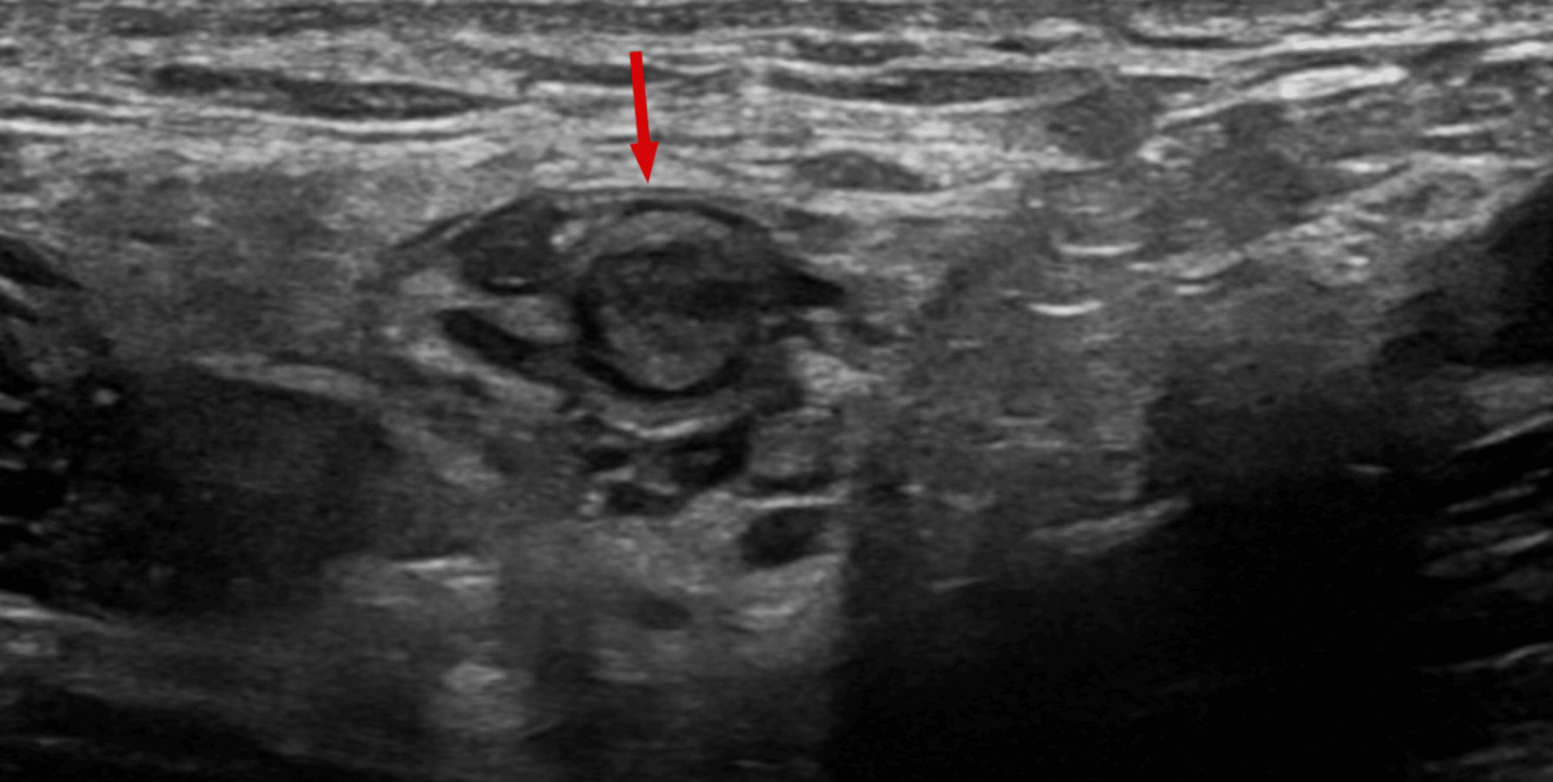
Transverse image of the left testicular vein at the level of the superficial ring of the inguinal canal. The vein is distended with thrombus and is not compressible. The arrow indicates the intraluminal thrombus within the distended left testicular vein.

**Figure 2:**
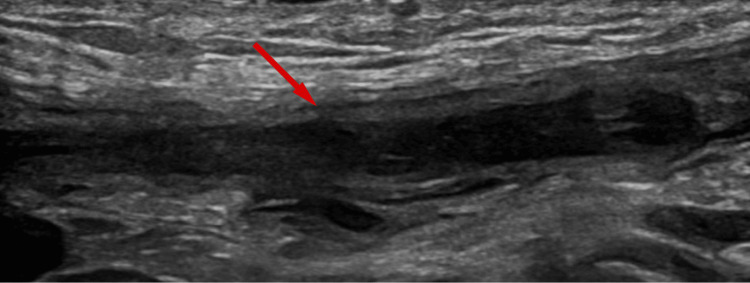
Longitudinal image of the left testicular vein in the inguinal canal. The vein is distended with a thrombus. The arrow indicates the intraluminal thrombus within the distended left testicular vein.

**Figure 3:**
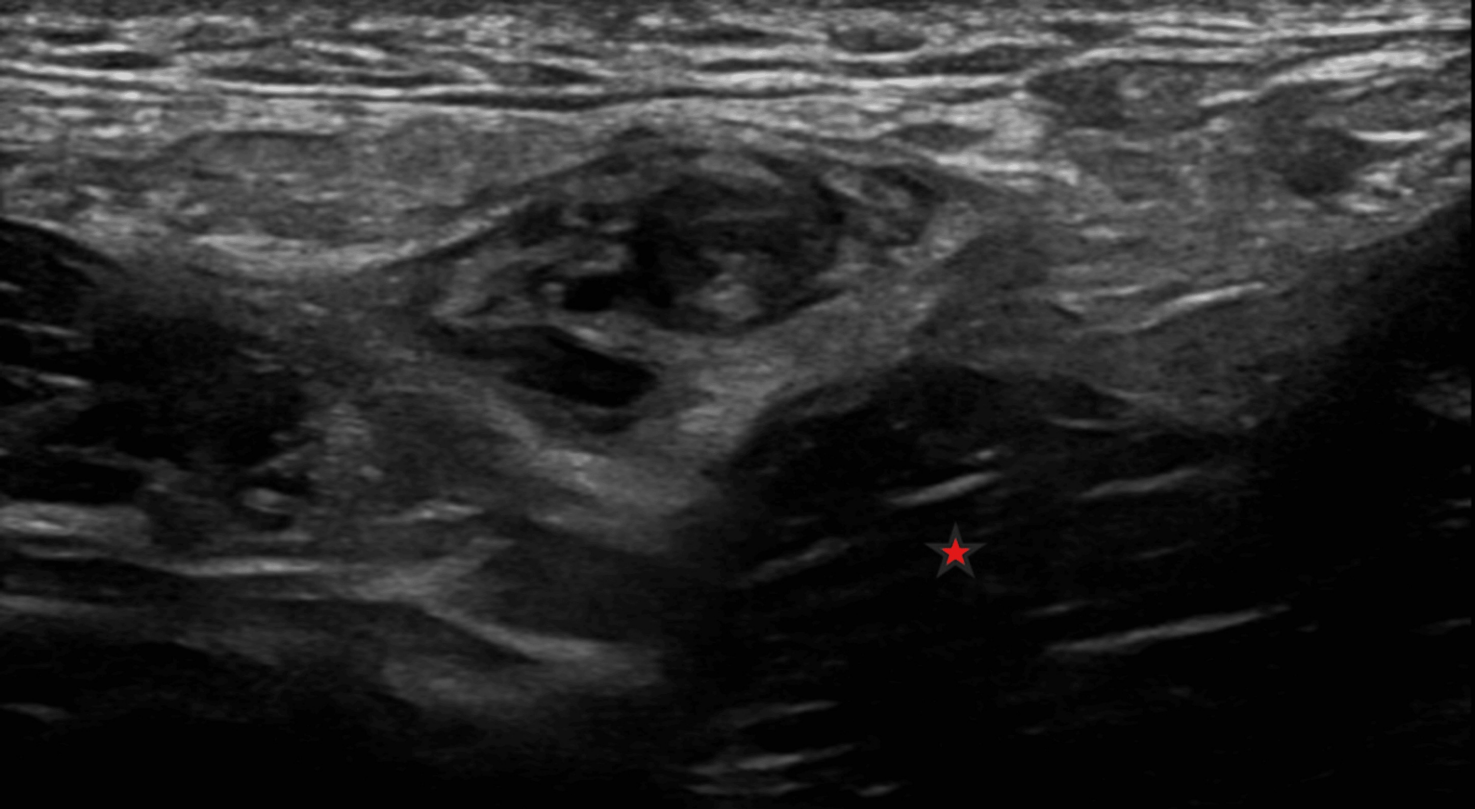
Transverse image of the left testicular vein in the lower abdomen, anterior to the left psoas muscle (red star). The vein is distended with thrombus and is not compressible.

Color Doppler ultrasound showed normal testes with preserved perfusion. The left testicular vein contained a non-compressible thrombus (Figures 1, 2) extending into the abdominal portion anterior to the psoas muscle (Figure 3). The left renal vein and the inferior vena cava (IVC) were patent.

**Figure 4:**
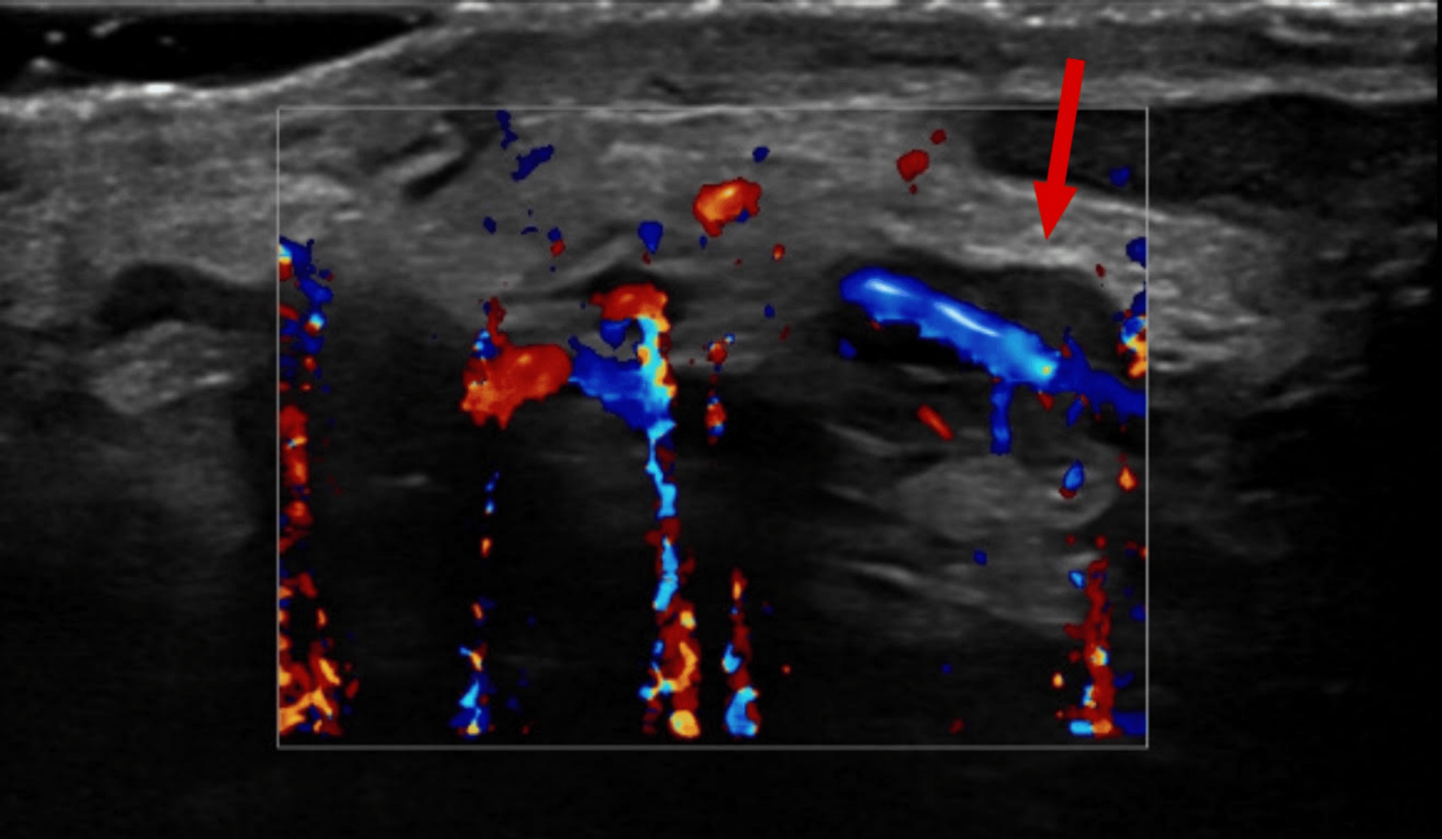
Interval partial recanalization of the previously thrombosed left testicular vein (arrow).

Apixaban was initiated on hematology advice for a short course of four weeks due to the localized nature of the thrombus. The patient was advised to restrict physical activity and use scrotal support. Follow-up Doppler at four weeks demonstrated no thrombus and partial recanalization of the left testicular vein, with resolution of symptoms (Figure 4).

No additional imaging or hematologic reassessment was scheduled, as the patient remained asymptomatic and hematology review did not identify any clinically significant abnormalities requiring further evaluation.

## Discussion

Testicular vein thrombosis is a rare cause of acute or sub-acute testicular pain and swelling, with only around 42 cases reported in the literature [1-4]. Most cases are idiopathic, with no identifiable risk factors such as trauma, infection, surgery, or systemic disease [4-7]. In our patient, a comprehensive hematological evaluation, including a targeted thrombophilia panel and lupus serology, revealed no clinically significant abnormalities.

The left testicular vein is more commonly involved, likely due to its longer course and higher hydrostatic pressure, which may predispose to venous stasis. Additional contributors, such as transient hypercoagulability or minor endothelial injury, can be considered within the framework of Virchow’s triad [1-3]. In this patient, no predisposing factors or identifiable cause for the thrombosis were found, consistent with an idiopathic presentation.

Clinical presentation is often non-specific and can mimic epididymitis, varicocele, testicular torsion, or inguinal hernia, making accurate diagnosis challenging [2,3,5]. Doppler ultrasound is the first-line imaging modality, allowing visualization of thrombus and assessment of venous flow [3,5]. CT angiography may be used to assess thrombus extension into the abdomen and evaluate the renal veins and IVC, as in our patient [2,8].

Management is typically conservative. Short-term anticoagulation, activity modification, and scrotal support are generally sufficient, while surgical intervention is rarely required [4,5,7]. Hematology involvement is essential to guide anticoagulation duration and interpret thrombophilia results. Previous reports on testicular vein thrombosis have described various conservative approaches, including observation, supportive care, non-steroidal anti-inflammatory drugs, and anticoagulation depending on thrombus extent and patient symptoms [4,5,8]. In our patient, apixaban for four weeks, together with activity restriction and scrotal support, resulted in partial recanalization and complete symptomatic resolution, without the need for further follow-up imaging, as no additional thrombus or complications were identified.

Follow-up imaging is recommended to ensure thrombus resolution and guide further management [3,8]. Most reported cases show favorable outcomes with conservative treatment, emphasizing the importance of early recognition to avoid unnecessary surgical intervention [1,4,7].

## Conclusions

Testicular vein thrombosis should be considered in young patients presenting with acute testicular pain and swelling. Early recognition with Doppler ultrasound is essential to guide management. Short-term anticoagulation, activity modification, and scrotal support are effective and generally lead to favorable outcomes. Close follow-up with imaging ensures thrombus resolution and prevents complications. Awareness of this condition allows clinicians to avoid unnecessary surgical interventions and provides reassurance regarding prognosis.
